# Effects of Proinflammatory Cytokines on Lacrimal Gland Myoepithelial Cells Contraction

**DOI:** 10.3389/fopht.2022.873486

**Published:** 2022-04-27

**Authors:** Angela Garriz, Junji Morokuma, Maytal Bowman, Sarah Pagni, Driss Zoukhri

**Affiliations:** ^1^Department of Comprehensive Care, Tufts University School of Dental Medicine, Boston, MA, United States; ^2^Public Health and Community Service, Tufts University School of Dental Medicine, Boston, MA, United States; ^3^Department of Ophthalmology, Tufts University School of Medicine, Boston, MA, United States

**Keywords:** contractile proteins, interleukin-1b, inflammation, myoepithelial cells, lacrimal gland supported by NHI R01EY029870

## Abstract

In the lacrimal gland, myoepithelial cells (MEC) express muscle contractile proteins such as alpha smooth muscle actin (SMA) and calponin and therefore can contract to help expel lacrimal fluid. In a previous study, we demonstrated that lacrimal gland MEC express the oxytocin receptor (OXTR) and they contract under oxytocin (OXT) stimulation. Using NOD and MRL/lpr mice (animal models of Sjogren’s syndrome), we reported a decrease in SMA and calponin protein levels plus a decline in acini contraction after stimulation with OXT. It is known that proinflammatory cytokines, such as interleukin-1β (IL-1β), tumor necrosis factor alpha (TNF-α) or interferon gamma (IFN-γ), can affect OXTR expression and signaling capacity and inhibit MEC contraction. The aim of the current study was to investigate if proinflammatory cytokines are implicated in the loss of MEC contractile ability. Thus, lacrimal gland MEC from SMA-GFP transgenic mice were treated with IL-1β (10 ng/ml) for a total of 7 days. At days 0, 2, 4 and 7, GFP intensity, cell size/area, contractile proteins amounts and MEC contraction were assessed. At day 0, control and treated cells showed no differences in GFP intensity and cell size. GFP intensity started to decrease in treated MEC at day 2 (20%; p=0.02), continuing after day 4 (25%; p=0.007) and 7 (30%; p=0.0001). Mean cell area was also reduced at day 2 (34%; p=0.0005), and after 4 (51%; p<0.0001) and 7 days (30%; p=0.0015). The contraction assay at day 2 showed a 70% decrease of contraction in treated MEC (p<0.0001), 73% (p<0.0001) at day 4 and 82% (p=0.0015) at day 7 when compared to control. Levels of contractile proteins were measured on day 7 showing a decrease in SMA and calponin amount in treated MEC compared with the control group (around 30%; p=0.0016 and p=0.0206; respectively). Similar results were observed when TNF-α and IFN-γ were added along with IL-1β. Taken together the present data and those from our previous studies with Sjogren’s syndrome mouse models, they strongly suggest that proinflammatory cytokines affect lacrimal gland MEC contractile ability that may account for the reduced tear secretion associated with Sjogren’s syndrome dry eye disease.

## Introduction

Lacrimal gland myoepithelial cells (MEC) form an extensively branched network surrounding the acinar and ductal cells of the lacrimal gland epithelium, being an important component of its secretory apparatus (1–3). MEC are able to contract helping to expel lacrimal fluid and they express muscle contractile proteins such as alpha smooth muscle actin (SMA) and calponin as well as epithelial markers (keratin 5 and 14) (3). They also express both muscarinic and purinergic receptors implying that MEC can respond to neural stimuli (4–6).

We have recently demonstrated that lacrimal gland MEC express the oxytocin receptor (OXTR) and they contract under oxytocin (OXT) stimulation (7, 8). The OXTR, like many other G protein coupled receptors (GPCRs), can be desensitized by prolonged agonist stimulation which can lead to a reduced signal transduction (9–11). Normal desensitization of GPCRs is commonly mediated through β-arrestin binding, however, under inflammatory conditions, OXTR down-regulation has also been observed (12, 13).

Proinflammatory cytokines, such as interleukin-1beta (IL-1β), tumor necrosis factor alpha (TNF-α) or interferon gamma (IFN-γ), have been shown to be key mediator in lacrimal gland inflammation associated with Sjogren’s syndrome affecting OXTR expression and signaling capacity and impair MEC contraction critically impeding lacrimal gland fluid release (14). In addition, it has been reported that IL-1β decreases OXTR mRNA levels and downregulates OXT binding capacity in myometrial and decidua human cell cultures (15–18).

In our previous study, using animal models of Sjogren’s syndrome (NOD and MRL/lpr mice), we reported a decrease in SMA and calponin protein levels plus a decline in acini contraction after stimulation with OXT (7). These results indicated that MEC function is impaired during chronic inflammation of the lacrimal gland. Thus, the aim of the current study was to investigate if proinflammatory cytokines are implicated in the loss of MEC contractile ability. Lacrimal gland MEC from a SMA-GFP transgenic mouse strain were treated with IL-1β (10 ng/ml) for a total of 7 days. At day 0, 2, 4 and 7, GFP intensity, cell area, contractile proteins amounts and MEC contraction were assessed. Our data show that chronic treatment with proinflammatory cytokines decreased GFP intensity, cell size, SMA and calponin protein expression and inhibited OXT-induced MEC contraction. Similar results were observed when TNF-α and IFN-γ were added along with IL-1β.

## Materials and Methods

### Cytokines, Chemicals, and Antibodies

Recombinant human IL-1β, recombinant human TNF-α and recombinant human IFN-γ were purchased from PEPROTECH (Rocky Hill, NJ). Oxytocin was purchased from Sigma-Aldrich (Saint Louis, MO). Cell media Dulbecco’s Modified Eagle (DMEM) and RPMI-1640 medium (Roswell Park Memorial Institute), collagenase type II, penicillin-streptomycin, L-glutamine, and fetal bovine serum (FBS) were from Gibco (Waltham, MA). TrypLE Express was from Invitrogen (Carlsbad, CA). Rabbit polyclonal antibody against αSMA (ab5694 at 1:400 dilution) and rabbit monoclonal antibody against calponin (ab46794 at 1:2000 dilution) were from Abcam (Waltham, MA). All secondary antibodies were from LI-COR (Lincoln, NE).

### Animals

All experiments described herein were performed in accordance with the Association for Research in Vision and Ophthalmology (ARVO) statement for the use of animals in ophthalmic and vision research and were approved by the Tufts Medical Center Institutional Animal Care and Use Committee. Mice were maintained in constant temperature rooms with fixed light/dark intervals of 12 h length and were fed ad libitum. To obtain MEC, SMA‐GFP mice (C57BL6)/*SMA^CreErt2^* strain was used for this study that was described by Yokota (19) and were a kind gift of Dr. Ivo Kalajzic (UConn Health, Farmington, CT). In these mice, the lacrimal gland MEC, which express SMA, are therefore labeled with GFP.

### Isolation and Propagation of Lacrimal Gland MEC

Four- to 6-week-old SMA-GFP mice were euthanized and the exorbital lacrimal glands were removed and minced into lobules for collagenase digestion using our previously described protocol (5, 8). Briefly, lacrimal glands were washed in cold DMEM, gently minced with a scalpel and forceps to prepare 2-3 mm lobules and placed in digestion media (1.5 mL/gland of DMEM and 1.65 mg/mL of collagenase type II). Samples were then incubated in a shaking water bath (37°C and 100 rpm) for 20-30 minutes. At regular 5 min intervals, lobules were gently pipetted, 10 times, through tips of decreasing diameter. Digested media was filtered through a sterile cell strainer (100 µm nylon mesh; Thermo Fisher Scientific, Waltham, MA), remaining tissue pushed through the mesh using the pipette tip, and collected cells washed with 1-2 mL DMEM. Cells were then centrifuged at 100 x *g* for 5 minutes, resuspended in complete RPMI-1640 medium supplemented with 10% fetal bovine serum, 2 mM L-glutamine, and 100 µg/mL penicillin-streptomycin and centrifuged again at 100 x *g* for 5 minutes. Pelleted cells were resuspended in 10 mL complete RPMI media, plated in 100 mm culture dishes (VWR, Radnor, PA) and placed in a 37°C incubator (5% CO_2_).

### Cytokine Treatment of Lacrimal Gland MEC

Lacrimal gland MEC were seeded in 6-well plates in 5% FBS RPMI Media. Confluent to sub-confluent MEC cultures were treated with 10 ng/ml of IL-1β alone or in combination with 10 ng/ml each of TNF-α and IFN-γ, for a total of 7 days. Every other day media was changed, and 4-5 pictures were randomly taken from each well to perform further analysis of GFP intensity and cell area measurements. In other experiments, cells were trypsinized to perform the contraction assay, as described below. After the last day of treatment (day 7), cells were lysed, and protein samples were prepared for western blotting studies, as described below.

### Image Analysis of GFP Intensity and Cell Size Measurements

Images of MEC cultures were taken on day 0 (before addition of cytokines) then 2, 4 and 7 days of cytokines treatment using a digital camera (SPOT Insight CMOS; SPOT Imaging, Sterling Heights, MI) mounted on an inverted light microscope (Eclipse TE2000-S; Nikon Instruments Inc., Melville, NY). Total GFP intensity was analyzed, in each of these time frames from control and treated MEC, using ImageJ/Fiji software (ImageJ 1.53, National Institutes of Health, USA). Analyzing GFP intensity is an indirect indicator of SMA protein levels since GFP expression in MEC is under the control of the SMA promoter. In addition, a minimum of 5 to 6 cells per photograph were selected to measure the cell area (µm^2^) using SPOT Advanced Imaging software (Version 5.6).

### SDS-PAGE and Western Blotting

At the end of the cytokine treatment, lacrimal gland MEC from treated and control groups were lysed in 0.2 mL ice-cold radio-immunoprecipitation assay (RIPA) buffer (10 mM Tris-HCl pH 7.4, 150 mM NaCl, 1 mM EDTA, 1% Triton X-100, 0.1% sodium deoxycholate, and 0.1% SDS supplemented with protease inhibitors). Cell lysates were centrifuged at 20,000 x *g* for 30 minutes and the supernatant collected. Proteins were separated by SDS-PAGE on NuPage 4–12% Bis-Tris gels in MOPS-SDS buffer (Invitrogen, Carlsbad, CA). Protein in the gels were transferred to nitrocellulose membranes using NuPage transfer buffer (Invitrogen) and processed for immunoblotting. After transfer, nitrocellulose membranes were stained with REVERT Total Protein Stain (LI-COR) following the manufacturer’s instructions and prior to being blocked using Odyssey blocking buffer (LI-COR) for 1 hour at room temperature. Membranes were then incubated overnight at 4°C with the appropriate primary antibody for SMA and calponin diluted in blocking buffer + 0.1% tween-20. Following washing with Tris-buffered saline + tween-20 (TBS; 50 mM Tris-HCl, 150 mM NaCl, 0.1% tween-20, pH 7.6) membranes were then incubated for 1 hour at room temperature with their appropriate secondary antibodies followed by detection on a LI-COR Odyssey Infrared Imager. Staining in each lane for REVERT total protein stain (see **Supplementary Material**), and band intensity for immunoblotting was quantified using the LI-COR Image Studio software (v.4.0). Western blot band quantifications were then normalized to the total amount of protein in each lane.

### MEC Contraction Assay

On days 2, 4 or 7, cells were trypsinized from the 6-well plate and seeded overnight in a 24-well-plate, at a density of around 5,000 cells/well. Cells treated with cytokines and untreated cells (control) were stimulated with OXT (10^-6^ M) for 20 minutes. Video recording of the contraction process was performed using a digital camera (SPOT Insight CMOS; SPOT Imaging, Sterling Heights, MI) mounted on an inverted fluorescence microscope (Eclipse TE2000-S; Nikon Instruments Inc., Melville, NY). Also, still images were taken before and 20 min after OXT stimulation for image analyses. At least 10 random cells from each well and each condition were used for image analyses, using ImageJ/Fiji software (ImageJ 1.53, National Institutes of Health, USA). The perimeter of the same cell was calculated before and after OXT stimulation, and the difference between these two values that represents the decrease in cell size after OXT stimulation, was expressed in percentage.

### Statistical Analyses and Data Presentation

Statistical analyses were performed using GraphPad Prism Software (version 9.0; San Diego, CA). Where appropriate, data are presented as means ± standard deviation (SD). Data consisting of 2 groups were analyzed using a 2-tailed unpaired Student’s *t*-test or the Mann-Whitney U test for non-normally distributed data with significant results being considered at p-value < 0.05.

## Results

### Effect of IL-1β on GFP Intensity and MEC Size

**Figure 1:**
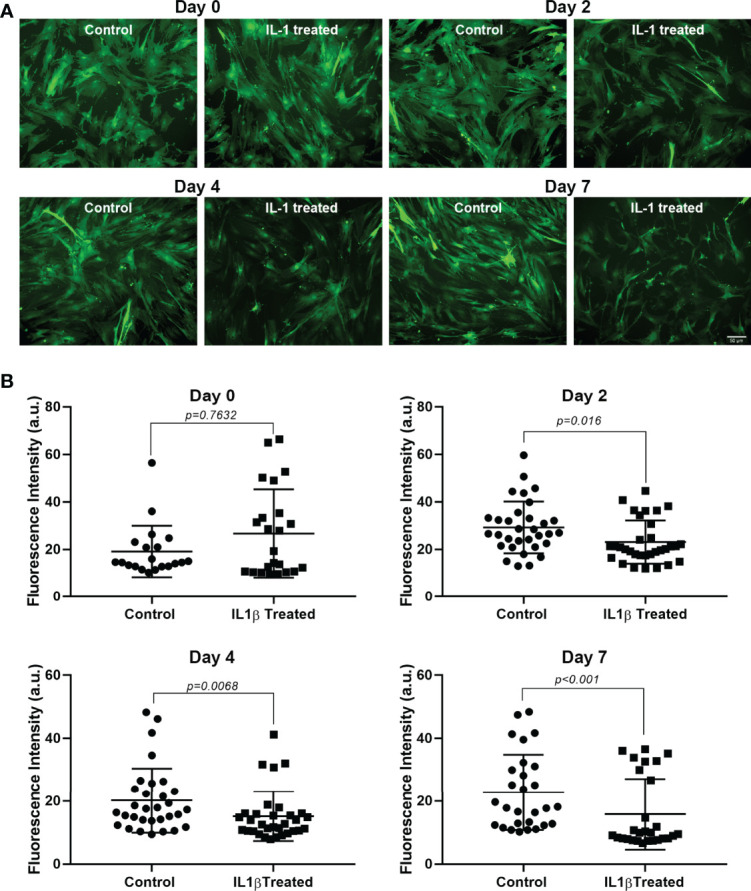
Effect of IL-1β on GFP intensity. Lacrimal gland MEC were either left untreated (control) of incubated with IL-1β (10 ng/ml) for 2, 4, or 7 days. Three or 4 random images were taken, using the same camera setting for all conditions, from each well and GFP intensity was quantified using ImageJ/Fiji software, as described in the Methods section. **(A)** Shows representative images from control and treated MEC at all time points measured and **(B)** Shows averaged data from 4 independent experiments. Compared to the control group, IL-1β treatment significantly decreased GFP intensity at all time points measured (Mann-Whitney U test). Data are means ± SD; n=20-23 for day 0; n=32 for day 2; n=31-32 for day 4; and n=28 for day 7 with all data from 4 independent experiments. Scale bar = 50 μm.

Since GFP expression in the transgenic mouse used in our studies is under the control of the SMA promoter, a decrease in GFP intensity would imply a decrease in SMA expression. Lacrimal gland MEC were left untreated or incubated with IL-1β for up to 7 days. Images were taken at days 0, 2, 4, and 7 and used for image analyses to quantify GFP intensity or MEC size, as described in the Methods section. As shown in **Figure 1**, at day 0, control and treated cells showed no difference in GFP intensity. GFP intensity started to decrease in treated MEC at day 2 (20%; p=0.02), continuing at day 4 (25%; p=0.007) and day 7 (30%; p=0.0001) (**Figure 1**).

**Figure 2:**
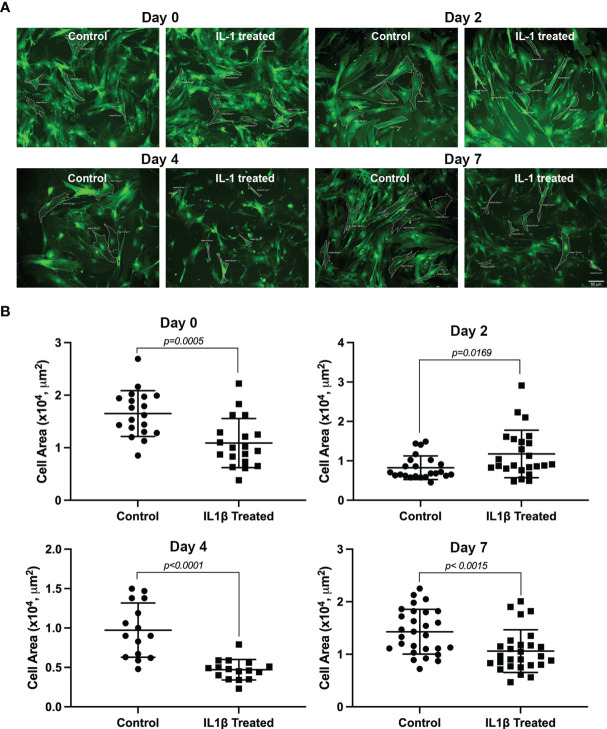
Effect of IL-1β on MEC size. Lacrimal gland MEC were either left untreated (control) of incubated with IL-1β (10 ng/ml) for 2, 4, or 7 days. Three or 4 random images were taken, using the same camera setting for all conditions, from each well MEC size was quantified using SPOT Imaging software, as described in the Methods section. **(A)** Shows representative images from control and treated MEC at all time points measured and **(B)** Shows averaged data from 4 independent experiments. Compared to the control group, IL-1β treatment significantly decreased MEC size at all time points measured (Student’s *t*-test). Data are means ± SD; n=23-24 for day 0; n=19 for day 2; n=15-16 for day 4; and n=27-28 for day 7 with all data from 4 independent experiments. Scale bar = 50 μm.

The data in **Figure 2** summarizes the effect of IL-1β treatment on MEC size. At day 0 there was no significant difference in cell size between the treated and control group. However, mean cell area was reduced at day 2 (34%; p=0.0005), day 4 (51%; p<0.0001) and day 7 (30%; p=0.0015) in treated MEC treated compared with the control group (**Figure 2**).

These data suggest that chronic treatment of lacrimal gland MEC with IL-1β lead to degradation of SMA protein, which resulted in smaller sized cells. Quantification of SMA and calponin protein expression levels, as discussed below, lend support to this hypothesis.

### Effect of IL-1β Treatment on MEC Contractile Proteins Levels

**Figure 3:**
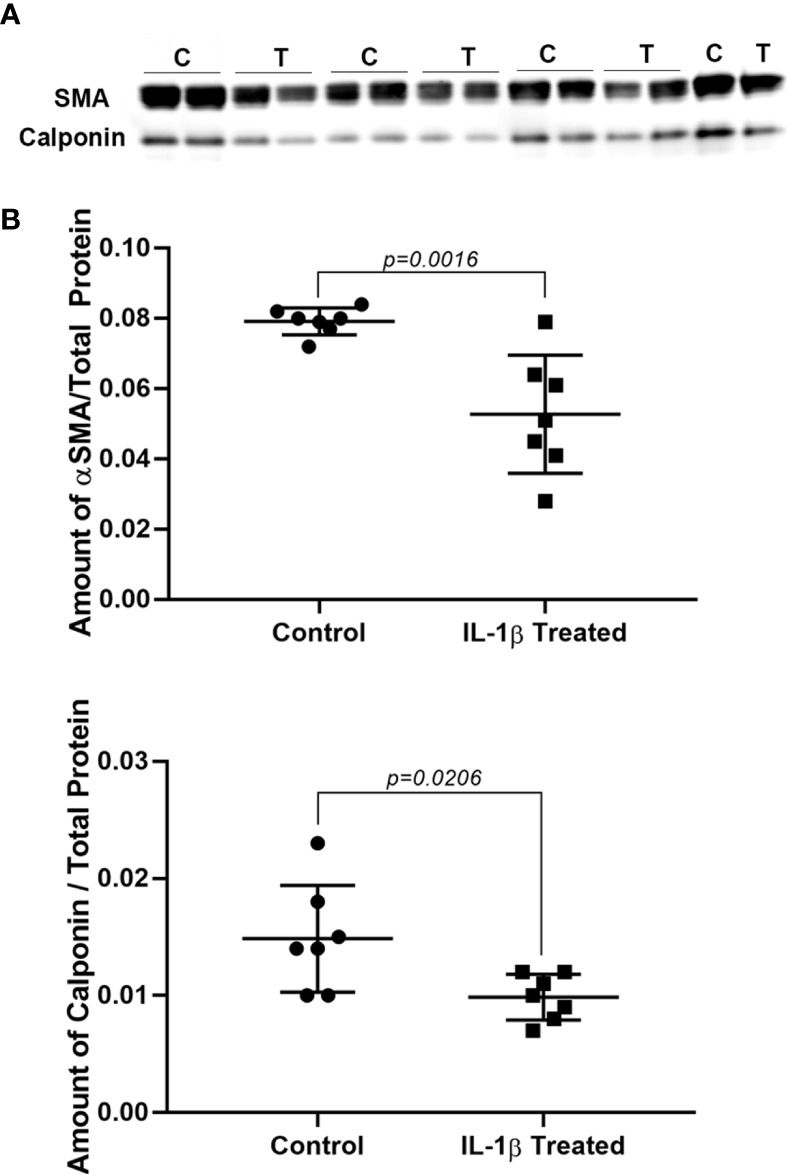
Effect of IL-1β on SMA and calponin protein levels. Lacrimal gland MEC were either left untreated (control) or treated with IL-1β (10 ng/ml) for 7 days. SMA and calponin protein level were quantified by western blotting and reported as a ration relative to total protein stain, as described in the Methods section. **(A)** Shows western blots for SMA (top) and calponin (bottom) in control **(B)** and IL-1β treated (T) MEC samples. **(B)** Graphs showing the amount of SMA and calponin in control and treated lacrimal gland MEC relative to total protein stain. SMA and calponin amounts are significantly decreased in lacrimal gland MEC treated with IL-1β compared with the control (*P* = 0.0016 and *P* = 0.0206; respectively, Student’s *t*-test.). Data in the plots are means ± SD, n = 7 from 4 independent experiments.

The data from the GFP intensity analyses suggested that chronic treatment of lacrimal gland MEC with IL-1β lead to lower expression of SMA protein. To test this hypothesis, we prepared cell lysates from control and IL-1β treated MEC and performed western blotting analyses to quantify the level of expression of SMA as well as calponin. As shown in **Figure 3**, there was a 33% decrease in the amount of SMA and calponin protein in treated MEC compared with the control group (p=0.0016 and p=0.0206; respectively). Please note that, as expected, SMA expression decreased to a similar level as did GFP intensity (**Figure 1**).

These data suggest that chronic treatment of MEC with IL-1β lead to lower expression of SMA and calponin proteins.

### Effect of IL-1β on OXT-Induced MEC Contraction

**Figure 4:**
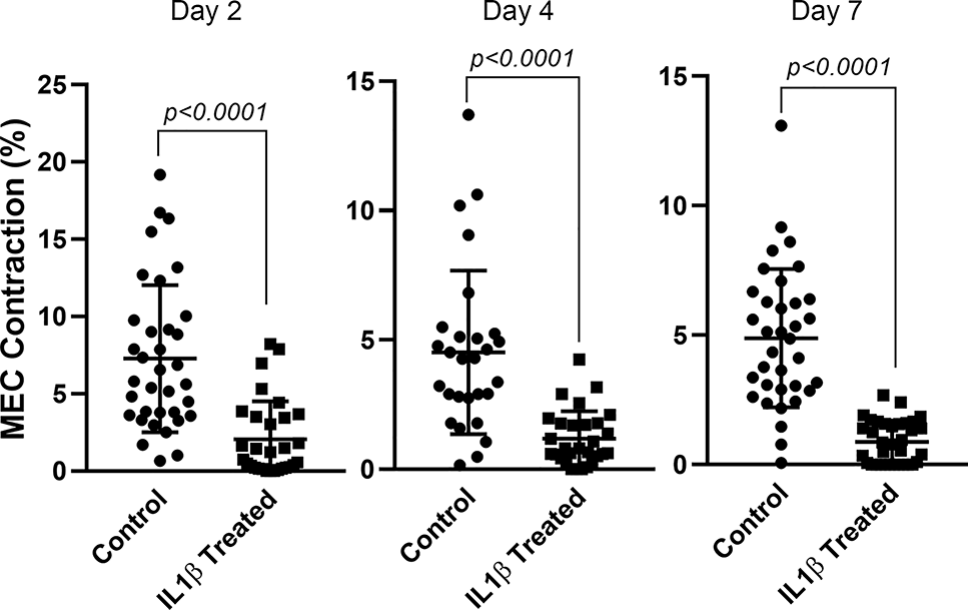
Effect of IL-1β on oxytocin-induced lacrimal gland MEC contraction. Lacrimal gland MEC were either left untreated (control) of incubated with IL-1β (10 ng/ml) for 2, 4, or 7 days. Cells were trypsinized for each time period, reseeded at low density and then stimulated with OXT (10^−6^ M) for 20 minutes. Changes in MEC size (i.e., contraction) following OXT stimulation was measured using ImageJ/Fiji software, as described in the Methods section. Chronic treatment of MEC with IL-1β significantly inhibited OXT-induced contraction at all three time points (Student’s *t*-test). Data are means ± SD; n=30-35 for day 2; n=28-29 for day 4 and n=34-35 for day 7 with all data from 3 independent experiments.

So far, our data suggest that chronic IL-1β treatment led to degradation of contractile proteins which could result in impaired lacrimal gland MEC contraction. Cells incubated with IL-1β for 2, 4 or 7 days were trypsinized, seeded overnight on 24-well plates to perform contraction assay as described in the Methods section. As shown in **Figure 4**, compared to controls, day 2 treated MEC showed a 70% (p<0.0001) decrease in OXT-induced contraction. Contraction was further decreased by 73% (p<0.0001) at day 4 and 82% (p=0.0015) at day 7 (**Figure 4**).

These data suggest that IL-1β induced lower expression of SMA and calponin proteins led to inhibition of OXT-induced MEC contraction.

### Effects of IL-1β, TNF-α and IFN-γ, on GFP Intensity, Cell Size and Contractile Protein Expression

**Figure 5:**
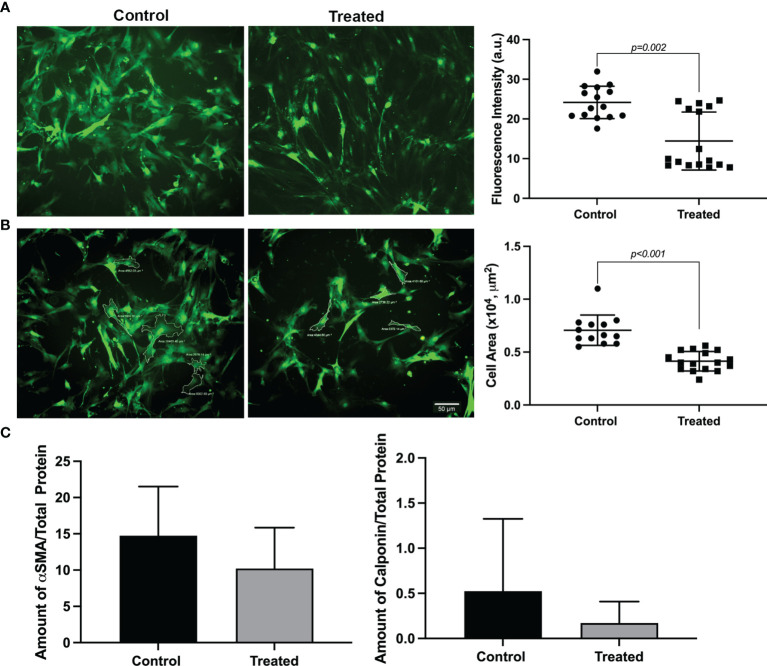
Effect of a cytokine cocktail on GFP intensity, MEC size and contractile protein expression. Lacrimal gland myoepithelial cells were treated for 7 days with 3 cytokines, IL-1β, TNF-α and IFN-γ, 10 ng/ml each. **(A)** Representative images of MEC at day 7 following cytokine treatment showing a decrease in GFP intensity. Scale bar = 50 μm. The graph next to the images shows a statistically significant decrease in GFP intensity in treated MEC compared with the control (*P* = 0.002; Mann-Whitney U test). Data are means ± SD, n=15-16 from 4 repeated experiments. **(B)** Representative images of MEC area measurements at day 7 following cytokine treatment showing a decrease in cell size. Scale bar = 50 μm. The graph next to the images shows a statistically significant decrease of MEC size in cytokine treated compared with the control (*P* < 0.001; Student’s *t*-test; data are means ± SD, n=13-14 from 4 independent experiments). **(C)** Graphs showing the amount of alpha smooth muscle actin (SMA) and calponin from MEC lysates treated with cytokines for 7 days compared with the control. The amount of both proteins tends to decrease, although not statistically significant (Mann-Whitney U test), in MEC treated with cytokines compared with the control. Data are means ± SD, n = 4.

The proinflammatory cytokines TNF-α and IFN-γ, are known to be elevated in chronically inflamed lacrimal glands as occurs in Sjogren’s syndrome. Therefore, we tested their effect, when added with IL-1β, on MEC functions. At day 0, GFP intensity and cell size of treated and untreated MEC was not significantly different (data non shown). In contrast, both GFP intensity and MEC size were significantly decreased following treatment with the cytokine cocktail for 7 days (**Figures 5A, B**). When compared to IL-1β alone, the cytokine cocktail decreased GFP intensity and cell size to a larger extent: 57% vs. 30% and 41% vs. 30%; respectively (**Figures 1**, **2**, and **5**). Similarly, the amounts of the contractile proteins, SMA and calponin, were also decreased, although not statistically significantly, following cytokine cocktail treatment (**Figure 5C**).

These data suggest that TNF-α and IFN-γ can synergize with IL-1β to further impact MEC functions in chronically inflamed lacrimal glands.

## Discussion

The data from the current studies show that chronic treatment of lacrimal gland MEC with the proinflammatory cytokine IL-1β led to lower expression of the contractile proteins, SMA and calponin, which resulted in smaller sized cells and inhibition of OXT-induced MEC contraction. These *in vitro* findings recapitulate our previously published *in vivo* findings in animal models of Sjogren’s syndrome with chronically inflamed lacrimal glands (7). Namely, the lower expression of SMA and calponin proteins, the reduced MEC size, and the loss of OXT-induced contraction.

It is known that several proinflammatory cytokines are highly expressed in Sjogren’s syndrome target organs (20–22). Some of the pro-inflammatory cytokines thought to play an important role in Sjogren’s syndrome pathophysiology are the interferons (IFN), IL-12, IL-18, TNF-α, IL-1β, IL-6 and B-cell activating factor (BAFF) (21). These cytokines are highly expressed in Sjogren’s syndrome with IFN in particular being responsible for activation of autoreactive T and B cells in the lacrimal glands (21, 23). In the current studies, addition of two other proinflammatory cytokines known to be associated with Sjogren’s syndrome pathophysiology, TNF-α and IFN-γ, seemed to potentiate (or synergize) the effects of IL-1β.

The mechanisms involved in proinflammatory cytokine-induced degradation of MEC contractile proteins and inhibition of OXT-induced contraction remain to be investigated. In several muscle tissues, studies have shown that both the OXTR as well as contractile proteins are down regulated by proinflammatory cytokines. For example, studies showed that proinflammatory cytokines, especially IL-1β, down-regulate the expression of the OXTR in uterine smooth muscle (15, 17). The effect of IL-1β was both at the mRNA level as well as the OXTR protein level, although the molecular mechanisms were not described (17). A study by Castro et al. (24) reported that matrix metalloproteinase 2 (MMP-2) interacts with calponin-1 in aortic vascular smooth muscle cells and that MMP-2 mediated proteolysis of calponin-1 during endotoxemia contributes to LPS-induced hypocontractility. It is worth noting that we showed that inhibition of MMP-2 leads to increased tear production in an animal model of Sjogren’s syndrome dry eye disease (25). Future studies testing the effect of MMP-2 inhibition on proinflammatory cytokine induced lacrimal gland MEC dysfunction are needed.

In myometrial cells, the activation of the transcriptional regulatory nuclear factor κB (NF-κB) family is the main component in IL-1β signaling cascade (26–28). Several studies showed a concerted increase in the expression of genes of the ubiquitin/proteasome pathway, including the muscle specific ubiquitin ligases Trim63 (MuRF-1), Fbxo32 (Atrogin-1), as well as many other 20S and 19S proteasome subunits and cathepsin L, activating as a final protein breakdown the ubiquitin pathway (29). In several disease conditions, calpain and/or caspase-3 were reported to mediate this initial breakdown (30–32). The role of these pathways in proinflammatory cytokine-mediated lacrimal gland MEC dysfunction remain to be investigated.

In conclusion, our data show that chronic treatment of lacrimal gland MEC with the proinflammatory cytokine IL-1β lead to lower expression of the contractile proteins SMA and calponin, reduced cell size and a profound inhibition of OXT-induced contraction. The addition of two other proinflammatory cytokines, TNF-α and IFN-γ, seemed to potentiate the effect of IL-1β on lacrimal gland MEC. These *in vitro* findings coupled with our published *in vivo* findings suggest that targeting proinflammatory cytokine in chronically inflamed lacrimal gland is a potential therapeutic target to restore MEC contractile ability and tear secretion in dry eye disease.

## Data Availability Statement

The raw data supporting the conclusions of this article will be made available by the authors, without undue reservation.

## Ethics Statement

The animal study was reviewed and approved by Tufts Medical Center Institutional Animal Care and Use Committee.

## Author Contributions

AG: Designed and performed experiments, analyzed data, wrote manuscript. JM: Performed experiments, analyzed data, wrote manuscript. MB: Performed experiments. SP: Analyzed data. DZ: Designed and performed experiments, analyzed data, wrote manuscript. All authors contributed to the article and approved the submitted version.

## Funding

Supported by NIH R01EY029870.

## Conflict of Interest

The authors declare that the research was conducted in the absence of any commercial or financial relationships that could be construed as a potential conflict of interest.

## Publisher’s Note

All claims expressed in this article are solely those of the authors and do not necessarily represent those of their affiliated organizations, or those of the publisher, the editors and the reviewers. Any product that may be evaluated in this article, or claim that may be made by its manufacturer, is not guaranteed or endorsed by the publisher.
